# Two patients with ZAP-70 deficiency in China present with a different genetic, immunological, and clinical phenotype

**DOI:** 10.1186/s12887-023-03975-6

**Published:** 2023-04-26

**Authors:** Xianze Luo, Qing Liu, Lina Zhou, Xuemei Tang, Xiaodong Zhao, Zhiyong Zhang

**Affiliations:** Department of Rheumatology and Immunology, Children’s Hospital of Chongqing Medical University, National Clinical Research Center for Child Health and Disorders, Ministry of Education Key Laboratory of Child Development and Disorders, Chongqing Key Laboratory of Child Infection and Immunity, Chongqing, China

**Keywords:** Combined immunodeficiency_1_, lymphoma_2_, Hematopoietic stem cell transplantation_3_, Primary Immunodeficiency_4_, ZAP-70 deficiency_5_

## Abstract

Zeta(ζ)-Chain Associated Protein Kinase 70 kDa (ZAP-70) deficiency is a rare autosomal recessive primary immunodeficiency disease. Little is known about this disease. In this study, we report two patients to extend the range of clinical phenotypes and immunophenotypes associated with ZAP-70 mutations. We describe the clinical, genetic, and immunological phenotypes of two patients with ZAP-70 deficiency in China, and the data are also compared with the literature. Case 1 presented with leaky severe combined immunodeficiency with low to the absence of CD8 + T cells, while case 2 suffered from a recurrent respiratory infection and had a past medical history of non-EBV-associated Hodgkin’s lymphoma. Sequencing revealed novel compound heterozygous mutations in ZAP-70 of these patients. Case 2 is the second ZAP-70 patient presenting a normal CD8 + T cell number. These two cases have been treated with hematopoietic stem cell transplantation. Selective CD8 + T cell loss is an essential feature of the immunophenotype of ZAP-70 deficiency patients, but there are exceptions. Hematopoietic stem cell transplantation can provide excellent long-term immune function and resolution of clinical problems.

## Introduction

Human inborn errors of immunity (IEI) are caused by monogenic germline mutations resulting in the loss or gain of function of the encoded protein. There are now 485 single-gene IEI and 10 main groups of the classification updated by the International Union of Immunological Societies in 2022, underlying phenotypes as diverse as infection, malignancy, allergy, autoimmunity, and autoinflammation [1].

Combined immunodeficiency (CID), the first IEI classification, is generally characterized by T cell development and/or functional defects [1]. It is well known that protein-tyrosine kinases (PTKs) play an integral role in T-cell activation. Activation of T cell antigen receptor (TCR) leads to tyrosine phosphorylation of several cellular proteins including Zeta(ζ)-Chain Associated Protein Kinase 70 kDa (ZAP-70), a member of the Syk family (non-receptor protein tyrosine kinase family), that co-precipitates with zeta upon TCR stimulation [2–4]. Activated ZAP-70 regulates motility, adhesion, and cytokine expression of specific lymphocytes. Thus, deficiency of ZAP-70 causes a CID phenotype, but slightly milder than those with recessive forms of severe CID (SCID) [5]. To date, 49 cases with ZAP-70 deficiency have been reported [6–17]. Patients with ZAP-70 deficiency often present a history of recurrent opportunistic infections, with autoimmunity, or immune dysregulation such as ulcerative colitis and cytopenia, pustular skin lesions, subcutaneous nodules, lymphoma, Omenn syndrome, and hemophagocytic lymphohistiocytosis (HLH). In 2017, we reported the first case of ZAP-70 deficiency in China (Qing Liu et al. 2016) [18]. So far, a total of two patients have been admitted to our cohort and been successfully treated with hematopoietic stem cell transplantation (HCT). Herein, we describe the characteristics of these two patients to extend the range of clinical, genetic, and immunological phenotypes associated with ZAP-70 mutations.

## Materials and methods

### Patients

Two patients with ZAP-70 deficiency were recruited from the Children’s Hospital of Chongqing Medical University in 2016 and 2020. Written informed consent was obtained from the patients’ families included in the study.

### Cells preparation

Peripheral blood mononuclear cells (PBMCs) were isolated from freshly drawn heparin-treated blood by Ficoll density gradient centrifugation.

### Immunological function analysis

#### Lymphocyte subsets

Whole blood was used for standard flow cytometry multicolor analysis; staining of lymphocyte surface markers was performed after red cell lysis, as described previously. A total of 20 subpopulations were examined to analyze the T and B lymphocyte subsets [19]. Also, Human Treg and Tfh cells were detected using monoclonal antibodies in PBMCs [18].

#### T cell receptor excision circle (TREC)

During T cell receptor rearrangement, excised DNA fragments create TREC. Quantification of TREC was performed by nested and quantitative real-time reverse transcription polymerase chain reaction (qRT-PCR), using DNA samples extracted from peripheral blood [20].

#### CDR3 spectratyping

Each T cell receptor (TCR) Vβ fragment was amplified using one of 23 Vβ-specific primers and a 5’FAM-labeled Cβ primer. The PCR products were sequenced by Sangon Biotech Company (Shanghai, China). The data were analyzed using Gene Mapper V3.5, and a scoring system was used to evaluate TCR Vβ diversity: a score < 4 indicated a skewed subfamily [21].

#### Proliferation of T cells and B cells

PBMCs were incubated with CFSE at 37 °C and then suspended in 600 µL of RPMI / 10% FBS and seeded into 96-well plates along with phytohemagglutinin (PHA), a lectin from pokeweed mitogen (PWM), and the same volume of RPMI for 72 h, as described previously. After staining of lymphocyte surface markers, cells were analyzed and examined by Flow cytometer [19].

### ZAP-70 expression

The expression of ZAP-70 in PBMCs was tested by Flow cytometer and Western blot with ZAP-70 antibodies (clone: 1E7.2, Biolegend) [18].

### Mutation analysis

Genetic testing of two patients was performed by Mygenostics company (Beijing, China). P1 was via panel and P2 was via whole external sequencing. No other suspected mutations were found except ZAP-70. In our laboratory, mutations were confirmed by Sanger sequencing [18]. Then, potential pathogenicity was evaluated using PolyPhen-2 (http://genetics.bwh.harvard.edu/pph2/index.shtml), MutationTaster (http://www.mutationtaster.org/), and PROVEAN (http://provean.jcvi.org/index.php). The potential structural impact of the novel mutations was predicted by SWISS-MODEL (https://swissmodel.expasy.org/) and PymoL 2.1 program (https://pymol.org/2/).

### Ethics approval

All procedures performed in studies involving human participants were in accordance with the ethical standards of the institutional and/or national research committee and with the 1964 Helsinki declaration and its later amendments or comparable ethical standards. The study described has been carried out under the abovementioned standards and has been approved by the Medical Ethics Committee of the Children’s Hospital of Chongqing Medical University.

## Results

### Clinical phenotype

Two patients were born at term to nonconsanguineous parents without a family history of immune-mediated diseases, respectively. They were vaccinated at birth with BCG with no significant adverse effects. The detailed clinical information has been summarized (Table 1).

**Table 1:** Clinical features of two patients with ZAP-70 deficiency

	P1	P2
Sex	Male	Female
Age of Onset	1 day	1 year
Age of Diagnosis	7 months	10 years
Diarrhea	Recurrent	-
Pneumonia	Recurrent	Recurrent
Sinusitis	-	Recurrent
Bronchiectasis	-	Yes, after 8 years old
Etiology of infections	(Sputum) *Klebsiella pneumoniae, syncytial virus*	(BALF) *Parainfluenza, Streptococcus pneumoniae, Haemophilus influenzae, Aspergillus, Syncytial virus, Pseudomonas aeruginosa*
Adverse reaction to BCG vaccination	-	-
Rash	Eczema	-
Hepatosplenomegaly	-	-
Autoimmunology	IBD	-
Malignancy	-	Hodgkin’s lymphoma of the groin at 5 years and recoverd after chemotherapy
Others	-	Febrile seizures
Outcome	HCT at 1 year, diarrhea was relieved and recovery was good	HCT at 10.5 years, cough was relieved and recovery was good

-, negetive; BALF, bronchoalveolar lavage fluid; IBD, inflammatory bowel disease; HCT, Hematopoietic stem cell transplantation

Case 1 (P1) had been described in our previous report [18]. This boy presented with leaky SCID and was characterized by early-onset recurrent diarrhea, pneumonia and eczema in early 2016, with a preliminary diagnosis of inflammatory bowel disease (IBD)-like manifestations. He received the HCT in December 2016 at one year old.

Case 2 (P2) was a 10-year-old girl who was admitted to our hospital in 2020 suffering from a recurrent respiratory tract infection. Bronchoscopy and lung CT suggested consolidation of lobe segments with partial atelectasis and the possibility of bronchiectasis. Etiological analysis for bronchoalveolar lavage fluid showed positive for parainfluenza virus, syncytial virus, aspergillus, Haemophilus influenza, and Pseudomonas aeruginosa, but negative for Epstein-Barr virus, cytomegalovirus, and Mycoplasma pneumonia. Sputum culture indicated streptococcus pneumonia (semi-quantitative 3+) and was positive for interferon tuberculosis. Moreover, she had a past medical history of non-EBV-associated classic Hodgkin lymphoma-mixed cell type IIIA (inguinal region) diagnosed by pathological biopsy at the age of 5 years and had received radiotherapy and chemotherapy for more than 1 year (The standard chemotherapy protocol was Cycle A - COPP/ABV - Cycle C - cycle A- COPP/ABV - Cycle C for 6 cycles). During the period of CT/RT, adverse events of peripheral blood cytopenia and pulmonary infections occurred. Also, she had two febrile convulsions. Blood routine results since age five indicated the presence of lymphocytopenia in P2. There was no diarrhea, abdominal distension, rash, pale face, epistaxis, oral ulcer, or skin abscess during her childhood. IVIG therapy was received after the identification of ZAP-70 deficiency, and HCT in March 2021 at 10.5 years old.

Before the diagnosis of ZAP-70 deficiency, two patients had received the BCG vaccine without adverse effects. P1 did not receive any treatment for tuberculosis since no evidence was detected. However, P2 could not rule out the diagnosis of tuberculosis infection because the T-SPOT test was positive and some enlarged lymph nodes were found in the lung CT, although pathogen analysis of tuberculosis using PCR and acid-fast staining was negative. To avoid suspicious tuberculosis infections, P2 received the treatment of ofloxacin, isoniazid, and rifampicin for one year after the diagnosis of ZAP-70 deficiency.

Two patients both received HCT with excellent outcomes. The conditioning treatment of P1 was BU + FLU + ATG for one week. Then 70ml mismatched (HLA 8/10) unrelated donor umbilical cord blood hematopoietic stem cells were reinfused, which achieved 11.48 × 10^8/Kg MNC (Mononuclear cells) and 16.74 × 10^6/Kg CD34 + cells. In P1, intestinal GVHD (Graft-versus-host-disease) and gastrointestinal bleeding were considered due to diarrhea and bloody stool after HCT. As a result, cyclosporine, mycophenolate, methotrexate, methylprednisolone, and tacrolimus were used. After the improvement of intestinal GVHD, prednisone combined with tacrolimus was adjusted, and the drugs were discontinued after one year. The conditioning treatment of P2 was BU + FLU + CTX for one week. Then 159ml matched (HLA 10/10) sibling donor peripheral blood hematopoietic stem cells from her sister were reinfused, which achieved 10.22 × 10^8/Kg MNC and 11.77 × 10^6/Kg CD34 + cells. After HCT, cyclosporine and mycophenolate were used to prevent GVHD. Methylprednisolone was also used because of suspicious implantation syndrome, and prednisone was changed after 2 weeks. Totally, mycophenolate was administered for 1 month, prednisone for 5 months, and cyclosporine for 1 year. GVHD did not occur in P2.

**Fig. 1:**
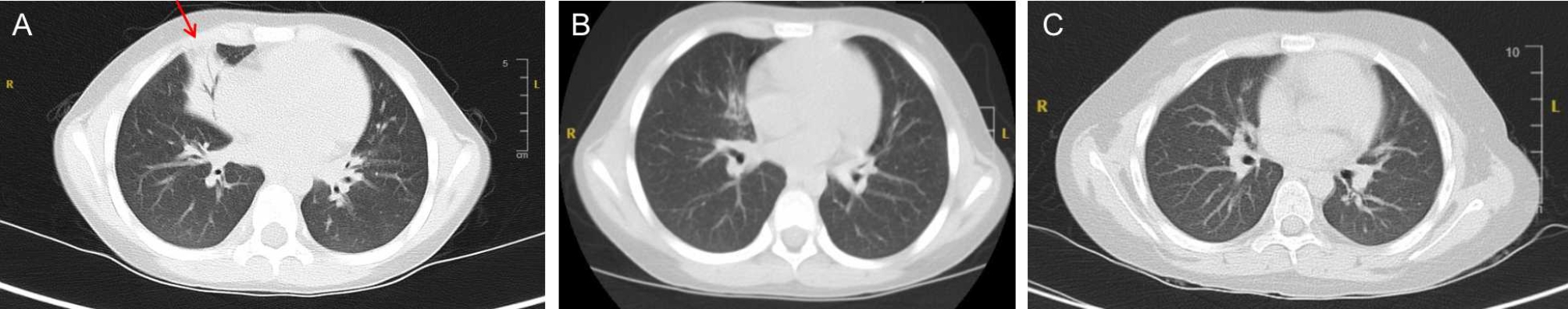
Lung CT of P2. The red arrow indicates lung inflammation. **(A)** suggests consolidation of the right middle lobe and left lower lobe segments with partial atelectasis (2018). **(B)** indicates less lung inflammation (3 months after IVIG therapy, 2020-11). **(C)** indicates no significant abnormalities (2 months after HCT, 2021-6)

Two patients achieved 100% chimerism one month later and remained stable in the follow-up. In addition, P1 no longer has recurrent diarrhea, now he grows well. The cough of P2 was relieved and the lung CT was completely normal (Fig. 1).The percentage and absolute count of B cells in P2 remained stable at normal levels 12 months later of HCT.

### Immunological phenotype

Flow cytometric analyses of the peripheral blood were performed for two patients. There was a decent reduction in the absolute number and percentage in CD8 + T cell (particularly, CD8 Naïve) in P1. However, this unique feature was not evident in P2 although she had lymphocytopenia. The absolute number of TCR γδ + T cell and B cell decreased, while NK cell counts were in the normal range. Before IgG substitution, lower levels of IgG, IgA, and IgM were detected in the serum of P1, but normal in P2. In both patients, the total IgE levels were always normal, far below the reference (150 IU/ML). The percentage of Tfh and Treg cell in the P1 was low (0.14% and 0.41%), but normal in P2 (14.2% and 8.79%). The hematological and immunological data of P2 are summarized (Table 2).

**Table 2:** A. Hematological and immune investigations of P2

Absolute count (reference range) Age(years)	5	6	8	10	11(6 months after HCT)	11(12 months after HCT)
WBC (×10^9^/L)(4.30–11.30)	10.33	5.84	7.14	5.91	9.35	7.38
Hb (g/L)(118–156)	128	124	152	136	128	129
PLT (×10^9^/L)(100–453)	391	209	245	263	123	250
Neutrophil (×10^9^/L)(1.60–7.80)	8.68	4.03	5.21	4.55	7.01	3.94
Lymphocyte (×10^9^/L)(1.50–4.60)	1.45	1.46	1.57	1.01	2.06	2.99
Total CD3 + T cells (×10^9^/L)(700–2100)	-	-	785	781	1819	1799
CD3 + CD4 + T cells (×10^9^/L)(300–1400)	-	-	501	377	145	425
CD3 + CD8 + T cells (×10^9^/L)(200–900)	-	-	282	332	1400	1200
NK cells (×10^9^/L)(90–600)	-	-	299	322	322	396
B cells (×10^9^/L)(100–500)	-	-	390	225	225	818
IgG (g/L)(5.28–21.90)	-	-	9.49	8.82	11.2	12.2
IgA (g/L)(0.440–3.950)	-	-	0.526	0.417	0.491	0.505
IgM (g/L)(0.48–2.26)	-	-	0.672	0.617	0.49	1.91
IgE (IU/mL)(0-165)	-	-	91	49	7.1	11.7

HCT, Hematopoietic stem cell transplantation. Reference range were presented with mean ± two standard deviations.

**Table 2:** B. Lymphocyte subsets analysis of P2 (10 years old)

	Absolute count (/µL)	Percentage (%)
T cells	588.0 (1297.0-2480.0)	58.8 (62.1–76.5)
CD8 + T cells	253.0 (508.7-1050.1)	25.3 (22.5–32.4)
CD8 + naïve	55.2 (232.0-665.1)	21.8 (36.1–72.3)
CD8 + TEMRA	13.3 (10.6-175.3)	5.2 (1.4–21.5)
CD8 + CM	172.8 (99.7-300.6)	68.3 (13.1–39.5)
CD8 + EM	11.7 (14.3-156.8)	4.6 (2.0-16.8)
CD4 + T cells	283.2 (621.4–1258.0)	28.3 (28.5–41.4)
CD4 + naïve	113.8 (299.0-857.0)	40.2 (39.9–71.8)
CD4 + TEMRA	1.1 (0.6–14.5)	0.4 (0.1–1.7)
CD4 + CM	148.4 (218.5-463.4)	52.4 (23.3–51.3)
CD4 + EM	20.0 (24.2–94.1)	7.1 (2.7–9.9)
TCR αβ + DNT	15.9 (11.8–41.4)	2.7 (0.7–2.2)
TCR γδ + T	24.8 (121.1-462.3)	4.2 (7.8–23.4)
B cells	169.3 (247.1-578.2)	16.9 (9.2–18.2)
Memory B	3.8 (29.5–89.7)	2.3 (8.9–22.9)
Naïve B	152.5 (140.3-380.9)	90.1 (45.0-75.8)
Transitional B	32.0 (5.1–36.6)	18.9 (1.8–10.3)
Plasmablasts B	0.6 (2.5–35.9)	0.3 (0.7-8.0)
NK cells	242.7 (202.5-583.5)	24.3 (7.8–23.5)

The proportions of CD4 + T cells, CD8 + T cells were calculated as percentages of total lymphocytes; the proportions of DNT cells and gdT cells were calculated as percentages of total T cells; subsets of CD4+, CD8 + T cells and B cells as percentages of CD4+, CD8 + T or B cells. Values of each population were presented with medians (upper line) and (10th to 90th) percentiles (lower line).

**Fig. 2:**
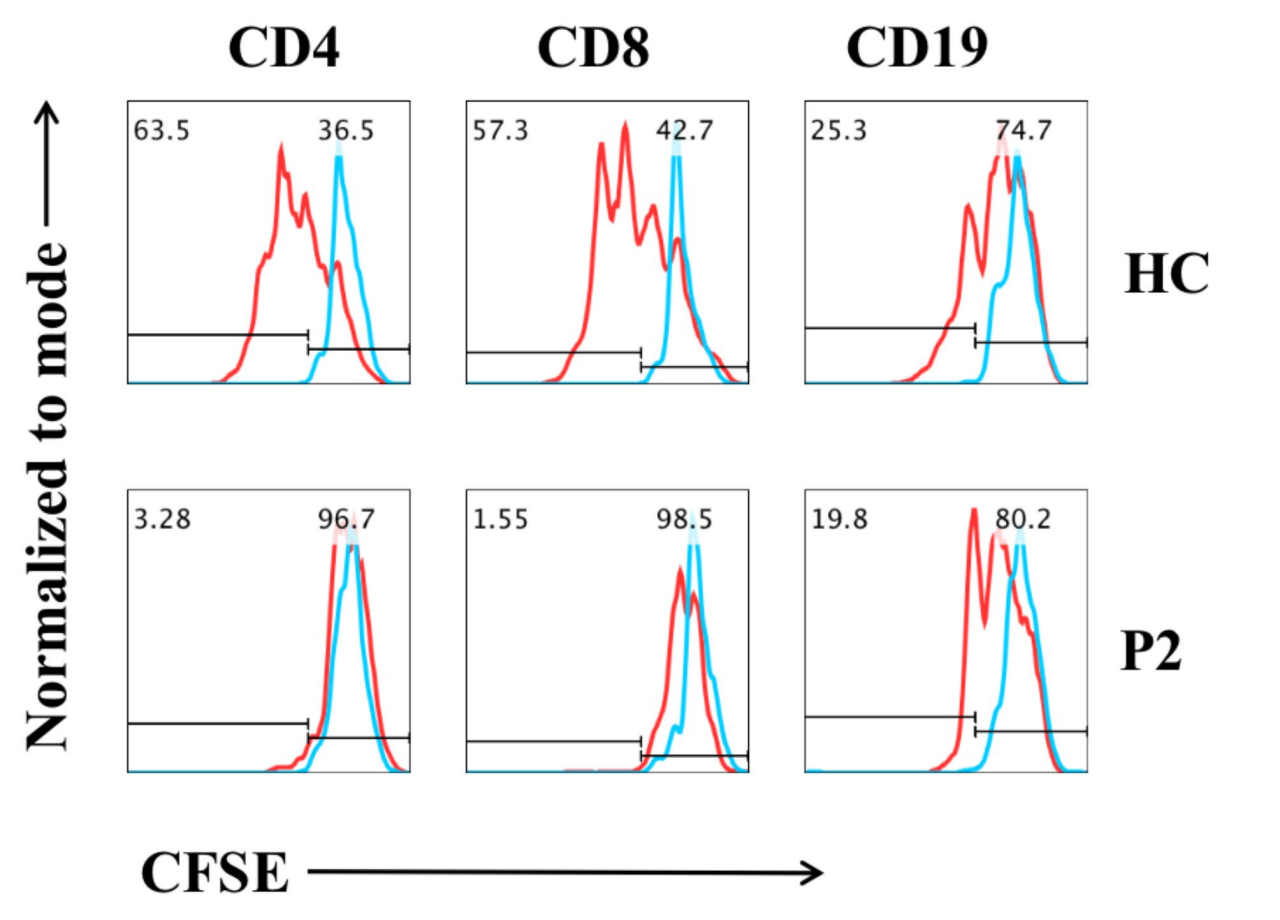
Flow cytometric analysis of the proliferation of P2 in vitro

Lymphocyte proliferation tests were performed in both patients. P1 had been described in our previous report, and the results of P2 were shown in Fig. 2. The percentage of CD4 + T cell and B cell proliferation of P1 was 0.31% and 1.04%, respectively, according to the lymphocyte proliferation test (the proliferation test could not carry out due in CD8 + T cell to the lack cells). The percentages of CD4+, CD8 + T cell and B cell proliferation in P2 were 3.28%, 1.55% and 19.8%, respectively, compared with 32.4% and 63.5%, 50.2% and 57.3%, 56.4% and 25.3%, respectively, in the normal control group.

**Fig. 3:**
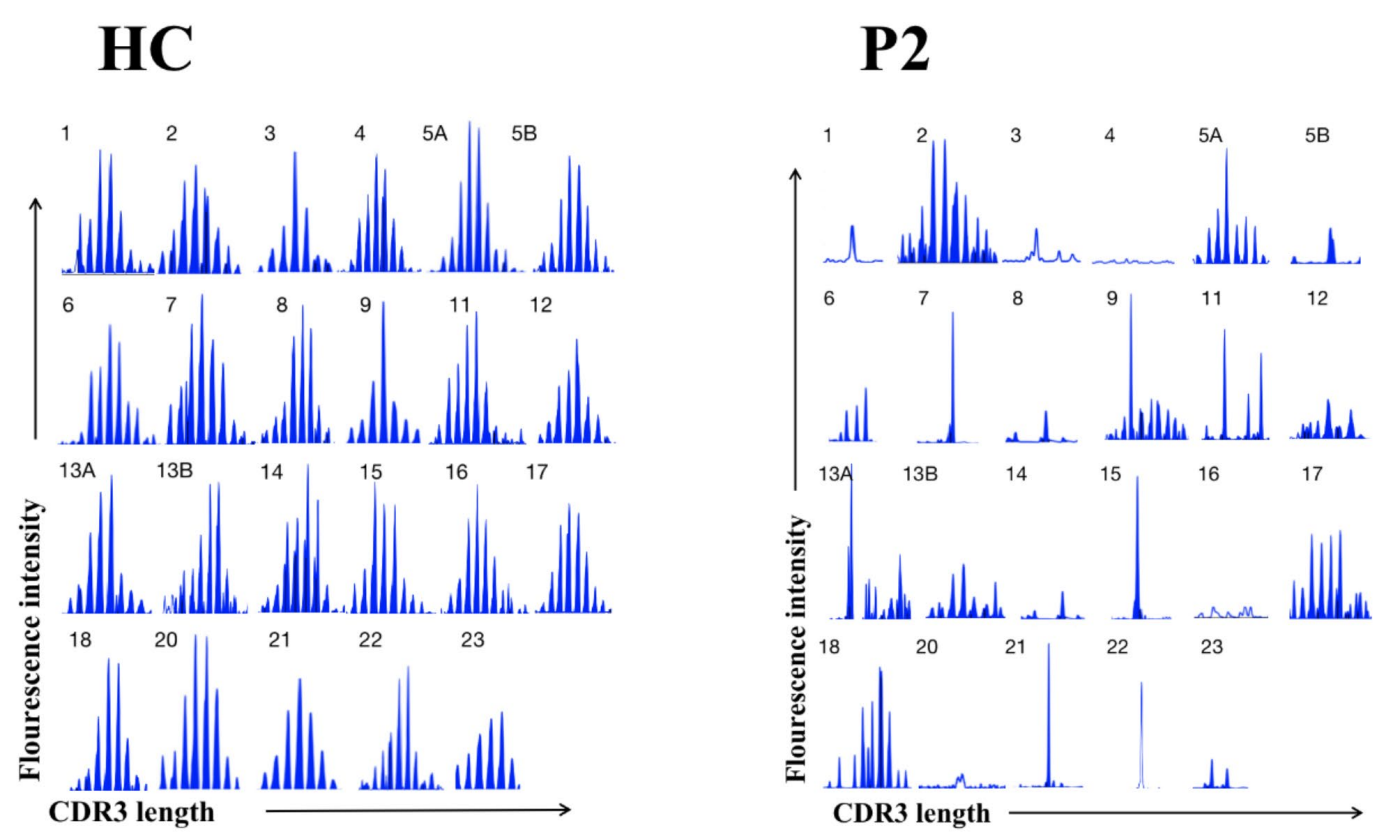
Analysis of TCR-Vβ diversity of P2 showed an oligoclonal peak, suggesting that TCR diversity was limited

TREC values of P1 and P2 were 17.5 and 10 copy number /ul, respectively, which were significantly lower than those of the normal age group. TCRVβ in P1 showed a polyclonal peak, while an oligoclonal peak in P2 (Fig. 3).

### Molecular characteristics

**Fig. 4:**
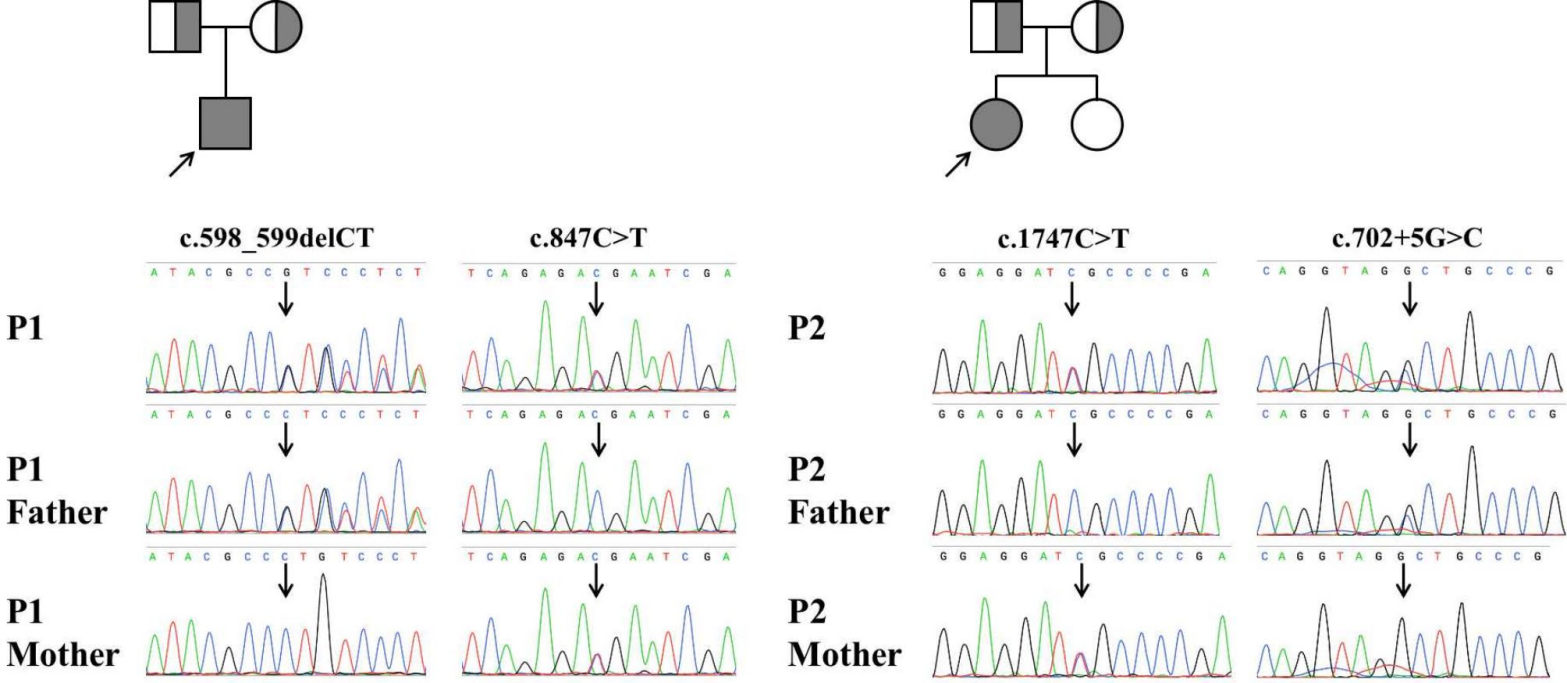
Pedigree and sequence analysis of ZAP-70 in the patients and their parents. Both patients were compound heterozygous mutations which were derived from parents, respectively

**Fig. 5:**
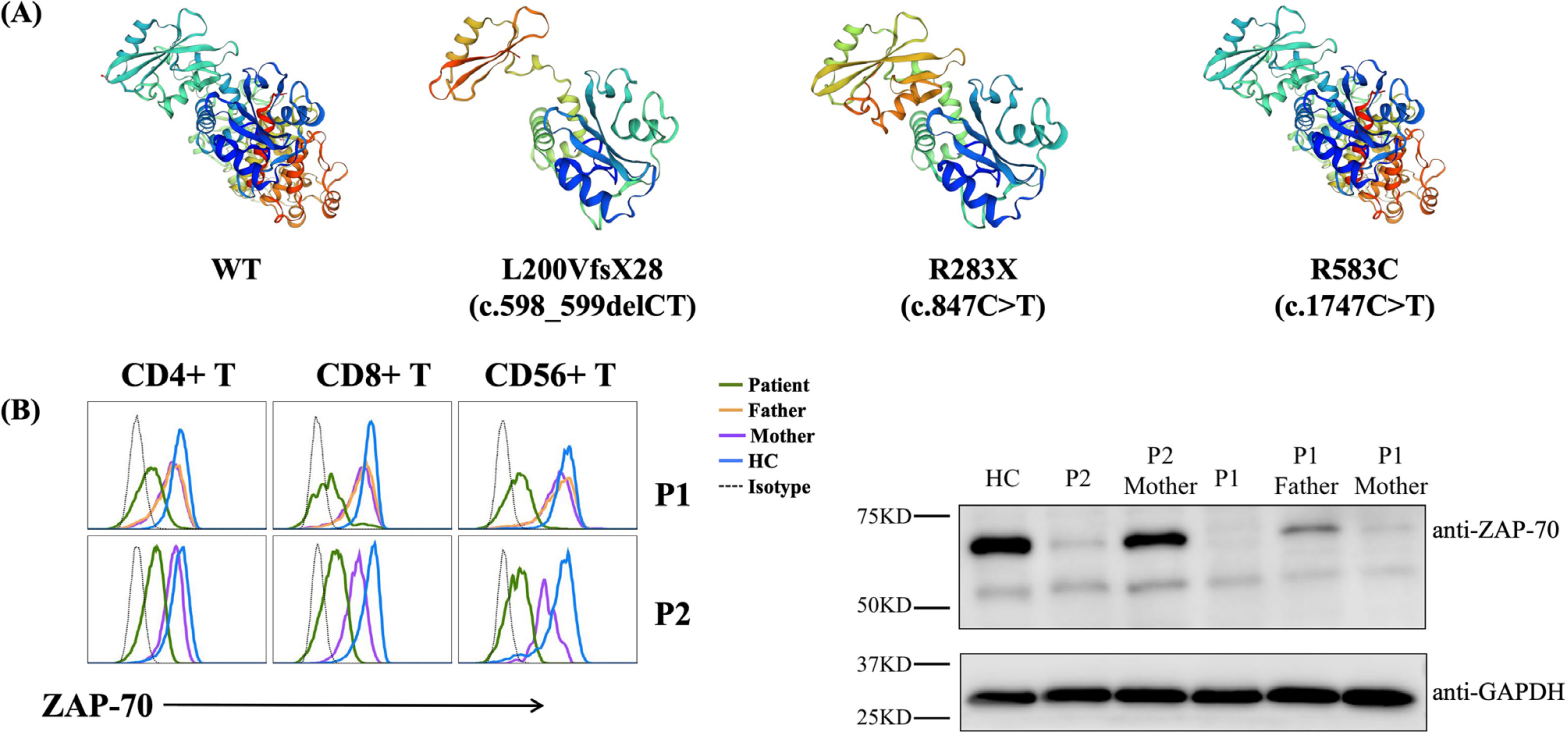
Structural characterization and expression of ZAP-70 protein. **(A)** WT (Wide Type) and mutations were drawn by SWISS-MODEL and PymoL. **(B)** Flow cytometric and western blot analysis of the ZAP-70 expression

Both patients were compound heterozygous for ZAP-70 (c.598_599delCT and c.847 C > T in P1; c.1747 C > T and c.702+5G > C in P2) which were derived from their parents, respectively (Fig. 4). Neither mutations of P2 had been reported previously, nor were found in the mbiobank (http://www.mbiobank.com/). Although there were no significant changes of p.R583C (c.1747 C > T) in protein structure predicted by SWISS-MODLE and PymoL, the mutant amino acid was predicted as pathogenic. Before HCT, flow cytometry indicated only residual ZAP-70 expression was identified in the patients. Evaluation of the parents revealed an intermediate ZAP-70 expression compared with that of the healthy control. Western blot showed that the expression of ZAP-70 was residual in P1, his parents, and P2, while the almost normal level in the mother of P2 (Fig. 5).

## Discussion

ZAP-70 deficiency is a rare autosomal recessive primary immunodeficiency disease with low to the absence of CD8 + T cell, most of the patients were initially diagnosed as SCID. To date, 49 cases are having been reported worldwide (P1 included). Most of the patients presented within 12 months of age with a median age of 4 months. However, the heterogeneity of clinical phenotypes and immunophenotypes is high and the cause is unclear [6]. In our study, P1 presented with leaky SCID while the clinical phenotype of P2 was more like antibody deficiency.

Respiratory tract infection which requires recurrent hospitalization was the most common first presenting feature followed by dermatitis in patients with ZAP-70 deficiency. The most common infection-causing agents identified were viruses (particularly CMV, Varicella, EBV, and Rotavirus), bacteria (mainly BCG), fungal pathogens (predominantly Candida albicans), and protozoa (especially Pneumocystis carinii). In our study, both patients presented recurrent pulmonary infection since infancy, but the pathogen was mostly bacteria [6]. P2, in particular, has experienced infection with such a limited pathogens spectrum so that the TCRVβ shows an oligoclonal peak. This may be related to delayed diagnosis and inadequate supportive treatment in our cohort. It also indicates the importance of antibiotics in the respiratory tract infection of these patients.Two patients had received the BCG vaccine after birth because it is one of the national immunization program vaccines in China. Luckily, no significant adverse effects happened. However, BCG is a unique pathogen associated with live attenuated vaccinations. Thus, a delayed BCG vaccination should be considered as a replacement for the live attenuated vaccination plan.

In addition to infectious complications, excessive inflammatory response and autoimmune diseases are also clinical features of ZAP-70 deficiency, including skin manifestations, hematologic abnormality, enteropathy, failure to thrive, lymphadenopathy, and malignancy [6]. In our study, the P1 child was accompanied by recurrent eczema and IBD but without failure to thrive. P2 developed bronchiectasis, which was considered to be related to chronic inflammatory stimulation and destruction of the airway. These non-infectious complications are considered to be due to the imbalance between T cell immune response and immune tolerance function caused by abnormal TCR signal transduction. This matches the reduction of the percentage of Tfh and Treg cell in P1.

Malignancy is another manifestation of ZAP-70 deficiency that cannot be ignored; three cases have been reported [15–17]. All of them occurred within one year of age, including EBV-associated diffuse large B-cell lymphoma, non-Hodgkin’s lymphoma, and a case of non-EBV-associated large B-cell lymphoma after HCT treatment. Notable, our patient P2 had a medical history of non-EBV-associated Hodgkin’s lymphoma at the age of five. It is considered the absence of CD8 + T cell (the cytotoxic and inhibitory T cell) increases the susceptibility to EBV; the decrease of TCR γδ + T cell increases the risk of malignancy. Besides, pustular skin lesions, subcutaneous nodules, Omenn syndrome, and hemophagocytic lymphohistiocytosis (HLH) have been reported in other ZAP-70 deficiency patients [6].

Low to the absence of CD8 + T cell is the most important feature of the immunophenotype of ZAP-70 deficiency patients. Studies have shown that only when TCR signal accumulation reaches a certain threshold, CD8 + single-positive T cell can survive and complete positive selection in the thymus [14]. Due to the deficiency of ZAP-70, TCR signal transduction was affected. Thus, CD4 + CD8 + pre-double positive T cell could not differentiate into mature CD8 + single-positive T cell, but only mature CD4 + T cell. In our study, a decent reduction in the absolute number and percentage in CD8 + T cell were only detected in P1 but not in P2. Notable, there was another patient with ZAP-70 deficiency found with normal CD8 + T cell number among the reported cases [8]. The clinical phenotype was recurrent infection and the genotype was a homozygous mutation of c.847 C > T (p.R283X). Thus, a normal CD8 + T cell count does not completely rule out this disease.

Possible explanations include maternofetal T cell transfusion, the less severe mutation sites, or the reverting mutations, that could improve the immunodeficiency of CID [22]. Unfortunately, that reported patient died at the age of one without further research, while our patient has been treated with HCT. There are no more opportunities to carry out further functional experiments or establish cell lines. Whether the lack of significant decline of CD8 + T cell is related to the short course of the disease or the residual expression of ZAP-70 remains to be explored. In fact, the deficiency of ZAP-70 affects not only the absolute number or percentage of CD8 + T cell but also in other subsets. The immunoglobulin level in P1 was significantly decreased, which was consistent with its B cell proliferation dysfunction. A marked decrease in Memory B cell and Plasma B cell were detected in P2 although the proliferation and immunoglobulin level was normal.

The pathogenicity of gene mutations has always been a challenge. The human ZAP-70 gene is located on chromosome position 2q11.2 that contains 2 non-coding and 12 coding exons. Structurally ZAP-70 is composed of two SH2 domains and a carboxy-terminal Kinase domain. Kinase domain is key to ZAP-70 activity while SH2 domains are coupled tightly and recognize phosphorylated immunoreceptor tyrosine-based activation motifs (ITAMs) with high specificity and affinity [23]. Most of the reported disease mutations in the Kinase domain abolished protein expression and/or disrupted catalytic activity. In Turkey, Iran, or Mennonite peoples, these mutation is usually homozygous because of consanguineous marriages and inbreeding [6]. In P1, we identified a nonsense and a frameshift mutation before the Kinase domain (SH2 and SH2-Kinase linker), resulting in a truncated protein expression and a classical phenotype. In contrast, P2 inherited novel compound heterozygous mutations, including a missense mutation located at the end of Kinase domain (c.1747 C > T, p.R583C) and a splicing mutation (c.702 + 5G > C), lead to a residual protein expression and less severe outcome. However, since the number of reported patients with ZAP-70 deficiency is small and the clinical pictures of the patients showed a striking heterogeneity, it is difficult to identify an association between the location and type of mutation with disease course.

Early diagnosis and timely treatment are key factors to improve the prognosis of IEI [1]. Parental consanguinity and family history are shown to be relevant indicators in the suspected patients. TREC detection has enabled the early diagnosis of many IEI with T cell defects as a neonatal PID screening tool. However, since the predominance of CD4 + T cell in neonatal T cell in the early stage, the current threshold level of TREC detection may not be able to detect ZAP-70 deficiency in the neonatal period [24]. While the attenuation of gene rearrangement in thymocytes was more obvious in ZAP-70 deficiency with age, it could be screened out during follow-up [25]. Thus, different screening thresholds for TREC may be considered in specific and high-risk ethnic groups, and TREC levels need to be followed up in patients with suspected ZAP-70 deficiency.

HCT is the only treatment for ZAP-70 deficiency and provides favorable long-term outcomes regardless of graft source [26, 27]. Our two patients with ZAP-70 deficiency received HCT at the age of one and 10.5 years, respectively. Acute GVHD was either minimal or absent and chronic GVHD did not develop. So far, their immune system has been reconstructed, and the outcome is satisfying. To date, 25 patients with ZAP-70 deficiency underwent HCT have been reported, with 91.7% surviving at a median follow-up of 36 months, compared to 59.1% at a median follow-up of 18 months for those without HCT. Among the 21 cases with donor type mentioned, 13 (61.9%) had a matched sibling donor and 8 (38.1%) had a matched unrelated donor. Besides, younger patients who undergo HCT experience better outcomes and fewer complications [6, 26]. Therefore, early screening and HCT could lower the burden of the disease.

Limitations to this study include the small sample size from a single center and the lack of further functional experiments. The expression of ZAP-70 protein in two patients has not been performed after HCT. Nevertheless, given the rarity of ZAP-70 deficiency, these two patients reported herein are still important for a clinical doctor to recognize this disease and make HCT decisions.

## Conclusion

In this paper, we have reported two rare cases of ZAP-70 deficiency and extend the genetic, immunological, and clinical spectrum of this disease. Patients with recurrent infection, combined with autoimmune or malignancy, especially those with selective CD8 + T cell loss, should be screened for ZAP-70 deficiency. However, there are exceptions that a normal CD8 + T cell count does not completely rule out this disease. HCT using a variety of graft sources a life-saving therapy for ZAP-70 deficiency provides excellent long-term immune function and resolution of clinical problems.

## Data Availability

The raw data and materials supporting the conclusions of this article will be made available by the authors, without undue reservation, to any qualified researcher.
